# Ingress of *Salmonella enterica* Typhimurium into Tomato Leaves through Hydathodes

**DOI:** 10.1371/journal.pone.0053470

**Published:** 2013-01-08

**Authors:** Ganyu Gu, Juan M. Cevallos-Cevallos, Ariena H. C. van Bruggen

**Affiliations:** Emerging Pathogens Institute and Department of Plant Pathology, University of Florida, Gainesville, Florida, United States of America; Charit-University Medicine Berlin, Germany

## Abstract

Internal contamination of *Salmonella* in plants is attracting increasing attention for food safety reasons. In this study, three different tomato cultivars “Florida Lanai”, “Crown Jewel”, “Ailsa Craig” and the transgenic line Sp5 of “Ailsa Craig” were inoculated with 1 µl GFP-labeled *Salmonella* Typhimurium through guttation droplets at concentrations of 10^9^ or 10^7^ CFU/ml. Survival of *Salmonella* on/in tomato leaves was detected by both direct plating and enrichment methods. *Salmonella* cells survived best on/in the inoculated leaves of cultivar “Ailsa Craig” and decreased fastest on/in “Florida Lanai” leaves. Increased guttation in the abscisic acid over-expressing Sp5 plants may have facilitated the entrance of *Salmonella* into leaves and the colonization on the surface of tomato leaves. Internalization of *Salmonella* Typhimurium in tomato leaves through guttation drop inoculation was confirmed by confocal laser microscopy. For the first time, convincing evidence is presented that *S. enterica* can enter tomato leaves through hydathodes and move into the vascular system, which may result in the internal translocation of the bacteria inside plants.

## Introduction

*Salmonella enterica* is one of the most common causal pathogens of enteric gastroenteritis worldwide [1]. Consumption of *Salmonella*-contaminated fresh produce, like tomatoes, has been reported to be associated with several multistate and international salmonellosis outbreaks in recent years [2]–[5].

Besides surface contamination through rain splash, aerosol or soil contamination [6]–[9], internalization of *Salmonella* spp. in plants, especially in lettuce and tomato plants, has been reported [10]–[12]. *S. enterica* Typhimurium can reach tomato fruit via internal translocation from leaves probably through phloem [11]. The chance of internal movement is low, but once *Salmonella* cells reach a fruit they can multiply to high densities within that fruit [11],[13].

Internalization of *Salmonella* into tomato leaves was reported to be possible through stomata [11]. However, hydathodes could be another or the main route for the entrance of this human pathogen into tomato plants. Hydathodes are stomata-like but permanently open water pores in intercellular spaces in the epidermis [14]. A small cavity is located underneath the hydathodes and above by epithem, which is composed of lose tissue that is closely associated with the ends of the vascular system in plant leaves. Under low transpiration and high soil moisture conditions, high root pressure of plants can secret water drops at the margin or tips of plant leaves through hydathodes, which is called guttation [15]. Carlton et al. showed that the bacterial canker pathogen, *Clavibacter michiganesis*, could enter tomato leaves and develop necrosis symptoms through hydathodes [16]. Norelli and Brandl reported that rapid temperature changes during summer storms could lead to the establishment of *Erwinia amylovora* (which belongs to the *Enterobacteriaceae*) in apple leaves through hydathodes and glandular trichomes [17].

The interactions between tomato plants and *Salmonella* may vary dependent on differences in plant cultivars and bacterial serovars or strains [13], [18]–[20]. However, the detailed mechanism for the colonization and survival of human pathogens in plants is still unknown. The plant hormone salicylic acid (SA) may affect biofilm formation of *Salmonella* Typhimurium [21], and colonization of the plant species *Medicago truncatula* by *Salmonella* Typhimurium results in the activation of SA-dependent and -independent plant defenses [22]. Abscisic acid (ABA), another important plant hormone, is involved in plant responses to environmental stresses, stomatal closure and developmental processes [23]. ABA biosynthesis can be regulated by 9-*cis*-epoxycarotenoid dioxygenase, which is encoded by the tomato *LeNCED1* gene [8]. Over-expression of *LeNCED1*in transgenic tomato plants leads to enhanced ABA biosynthesis and increased guttation [8], and possibly to increased ingress of pathogenic bacteria.

The purpose of this study was to determine whether hydathodes can serve as sites of entry for *Salmonella* and to investigate the effect of plant cultivar, guttation production and ABA on ingress and internal persistence of this pathogen in tomato plants.

## Methods

### Bacterial Strains and Plant Preparation

*S. enterica* Typhimurium strain MAE110, carrying kanamycin resistance and green fluorescent protein (GFP) genes on the chromosome, was provided by Dr. *Ute Römling* [24]. A bacterial culture was stored in Luria-Bertani (LB) broth containing 25% glycerol at −80°C. For each experiment, a loopful of the stored culture was added to shake cultures (150 rpm) of LB broth (50 µg/ml kanamycin), grown for 18 to 20 h at 37°C. The culture was harvested by centrifugation. The pellets were suspended in sterile distilled water (SDW) to a concentration of 10^9^ or 10^7^ CFU/ml for inoculation.

Tomato (*Solanum lycopersicum*) plants of three cultivars “Florida Lanai”, “Crown Jewel”, “Ailsa Craig” and one transgenic line of “Ailsa Craig”, Sp5, were used in this study. Transgenic tomato plant Sp5 with enhanced biosynthesis of plant abscisic acid and guttation [8] was generated by tranforming *LeNCED1* coding region under the chimaeric “Super-Promoter” into wild type “Ailsa Craig” [8]. Tomato seeds were surface disinfected with 1 M HCl for 30 min and germinated in potting mix. Two weeks after sowing seeds, seedlings were transplanted to sandy loam soil in 15-cm diameter pots. The sandy loam soil was collected from intensively managed research plots at the Plant Science Experiment Station of the University of Florida (Citra, Florida) where potatoes and corn were planted in 2009, peanuts and triticale in 2010, and soybeans in 2011 with typical fertilizer, fungicide, insecticide and herbicide application schedules. The contents of N, P, K and organic matter of the soil were 557, 85, 32 mg/kg and 1.33% (w/w), respectively, and the pH was 6.7. Water was applied at a 2-day interval, and fertilization was applied weekly with 150 ml half-strength Hoagland solution (pH 6.8). Plants were grown in a biological safety level 2 greenhouse equipped with ridge vents, a cooling air conditioning unit and a gas heater. The temperature fluctuated between 23°C and 33°C, with an average temperature of 28°C. Besides natural daylight, no additional lighting was provided. After 6–8 weeks, the tomato plants were moved from the greenhouse to a growth chamber (Percival Scientific, Inc., Perry, IA) for guttation formation and inoculation with *Salmonella*.

### Guttation Formation and Inoculation with *Salmonella*

In this study, the guttation inoculation and *Salmonella* detection experiments were conducted in triplicate to be considered as blocks in time. In each experiment, three plants of each tomato genotype (4 different genotypes) were randomly selected and inoculated with *Salmonella* Typhimurium strain MAE110 at one inoculation concentration (10^9^ or 10^7^ CFU/ml). The same numbers of plants were inoculated with 1 µL SDW as controls. Inoculated and control plants of the different genotypes were completely randomized inside the chamber.

Guttation was induced by changing the settings of the growth chamber to 14°C in darkness with 95% humidity for 10 h (Fig. S1). The volume of each guttation droplet was about 10 µL. Six terminal branches per plant were inoculated by transferring a 1 µL droplet of bacterial suspension (10^9^ or 10^7^ CFU/ml) or SDW (for control plants) to each of 4 guttation droplets on different leaflets per branch. After inoculation, the conditions in the growth chamber were adjusted to 28°C with 55% humidity during the day time (7 am to 9 pm), and 20°C with 75% humidity in the night. These are suitable conditions for *Salmonella* survival [25].The guttation droplets of inoculated plants were naturally drawn back into the leaf tissue within 3 h.

### Leaf Sampling and Testing Procedure

In each of the three replicate experiment, inoculated and non-inoculated leaflets were sampled 3 h, 1, 2, 3, 4, 5 days after inoculation with 1 µL of a10^9^ CFU/ml *S. enterica* Typhimurium suspension, and 3 h, 1, 2 days after inoculation with 1 µL of a 10^7^ CFU/ml suspension. At each sampling time, three inoculated leaflets of each plant were randomly removed. To wash off *Salmonella* cells attached on the surface of inoculated tomato leaflets, each collected leaflet was submerged in a 15 ml test tube with 5 ml SDW plus 0.1% (v/v) Tween 20 ™ (Sigma Chemical Co., St. Louis, MO), sonicated for 15 min in an Bransonic 5200 ultrasonic cleaner (Branson Ultrasonics Corp., Danbury, CT) and vortexed for 10 s. The washed off liquid was diluted 10-fold in phosphate buffered saline and 0.1 ml aliquots of the appropriate dilutions were spread over LB plates (50 µg/ml kanamycin). Thereafter, each leaflet was surface disinfected by 70% alcohol for 15 s, rinsed 3 times by SDW and ground in 1 ml SDW. The extract was plated on LB plates (50 µg/ml kanamycin) after preparing a 10-fold dilution series. One week after *Salmonella* inoculation with either dose, 4 inoculated leaflets from tomato plants of each genotype were randomly sampled, ground and enriched in LB broth (50 µg/ml kanamycin) at 37°C for 24 h. Numbers of *S. enterica* Typhimurium colonies on each Petri plate were determined by counting green fluorescent CFU’s using a UV lamp (UVGL-25, Entela Inc., USA). All plates were checked under UV light to exclude the possibility of counting colonies that were not the gfp-marked *Salmonella* strain. Very few unidentified bacterial colonies were found on the LB agar with kanamycin; these did not show green fluorescence under UV light.

### Microscopic Observations

In the first replicate of the experiments, two inoculated leaflets were sampled 1 day after the guttation inoculation at 10^9^ CFU/ml from each of the three inoculated plants of each genotype (six inoculated leaflets for each genotype). The tips of collected leaves were cut off for fluorescent microscopic analysis as described previously [11]. Forty leaf sections were checked for each leaflet. GFP-labeled *Salmonella* cells in the tissue sections were observed under a confocal laser scanning microscope (Olympus IX81-DSU; Olympus, Japan). The tissue sections were scanned for fluorescent bacteria under light with an excitation wavelength of 488 nm and a BA505-525 emission filter (GFP). Time lapse microscopy of a single field was employed to verify internal colonization of *Salmonella* cells. To confirm the absence of cross contamination and distinguish the presence of GFP labeled bacteria in inoculated leaves, equal numbers of leaf tips from control plants were checked under the microscope as described above.

### Statistical Analysis

The number of colonies per plate was converted to CFU/ml or CFU/g (fresh weight) and log-transformed to obtain normal distributions for statistical analysis. The cultivar effect of tomato plants on the survival of *Salmonella* on/in tomato leaves after guttation inoculation was evaluated by analyzing the *Salmonella* population on/in tomato leaves one day after inoculation by ANOVA or by fitting log-transformed data (separately for each replication) to the linear model (Y = aX+b) in Excel, in which Y = *S. enterica* Typhimurium concentration (log (CFU/g)), b = initial bacterial concentration (intercept, log (CFU/g)), a = decrease rate (slope, (log (CFU/g))·day^−1^) and X = time (days after inoculation). Estimated values of the parameters were subjected to multivariate analysis of variance (MANOVA). Similarly, log-transformed data of *Salmonella* concentrations on/in tomato leaves of transgenic Sp5 plants were compared with those of its wild type “Ailsa Craig”. Tomato genotypes and inoculation concentrations of *Salmonella* were considered as fixed-effect factors and repeats of the experiments (true replicates) were considered as blocks for statistical analysis. All statistical analyses (ANOVA, MANOVA, and t tests) were performed using SAS (SAS release 9.2, SAS Institute Inc., Cary, NC).

## Results

### Effect of Plant Cultivars on the Internalization and Survival of *Salmonella* Typhimurium in Tomato Leaves Inoculated through Guttation Droplets

**Figure 1:**
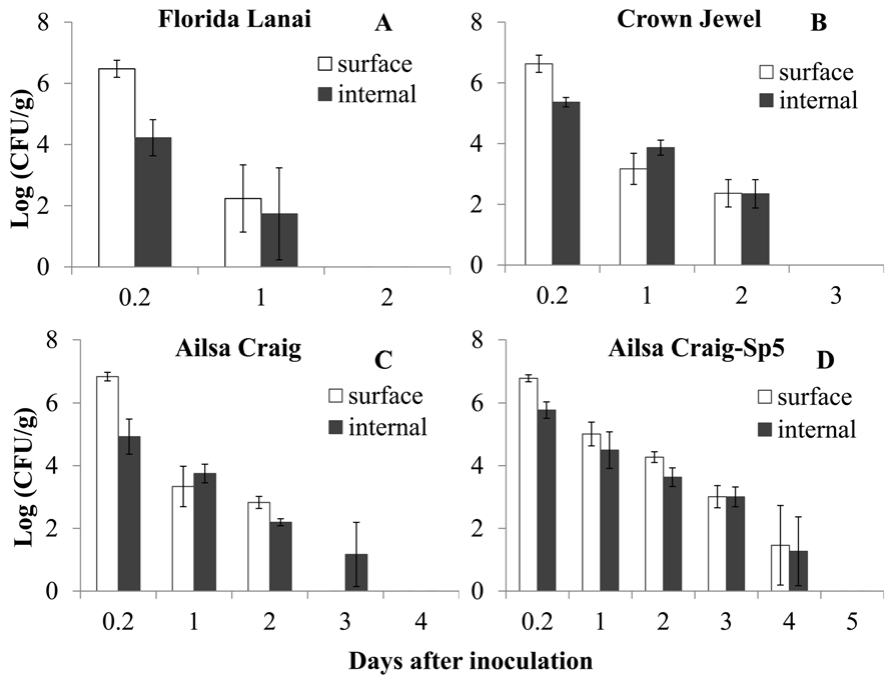
Survival of *Salmonella* Typhimurium on/in tomato leaves after inoculation (10^9^ CFU/ml) into guttation droplets. Populations of *Salmonella* (log(CFU/g)) in inoculated tomato leaves of tomato cultivar: Florida Lanai (A), Crown Jewel (B), Ailsa Craig (C) and the transgenic line (Sp5) of Ailsa Craig (D).

**Figure 2:**
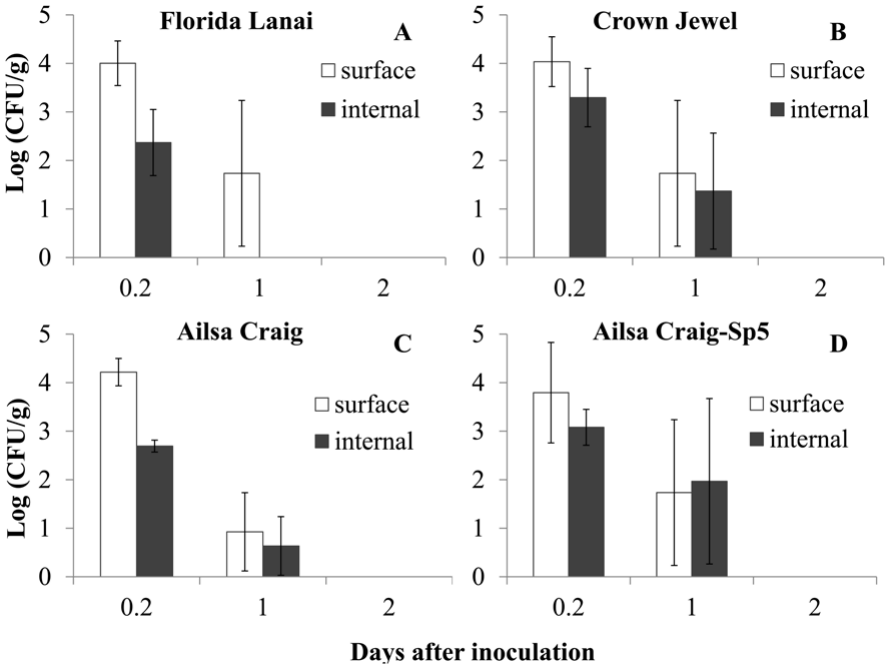
Survival of *Salmonella* Typhimurium on/in tomato leaves after inoculation (10^7^ CFU/ml) into guttation droplets. Populations of *Salmonella* (log(CFU/g)) in inoculated tomato leaves of tomato cultivar: Florida Lanai (A), Crown Jewel (B), Ailsa Craig (C) and the transgenic line (Sp5) of Ailsa Craig (D).

After guttation inoculation, *Salmonella* was detected inside the leaves as well as on the surface of leaves. The bacterial population decreased on/in leaves of the 3 different cultivars of tomato plants at both inoculation concentrations (Fig. 1 and Fig. 2). There were significant interactions between plant cultivars and inoculation concentrations for both *Salmonella* populations 1 day after inoculation and decline rates on/in tomato leaves (P<0.05).

At the inoculum concentration of 10^9^ CFU/ml, *Salmonella* in tomato leaves could not be detected 2 days after inoculation for cultivar “Florida Lanai”, 3 days for cultivar “Crown Jewel” and 4 days for cultivar “Ailsa Craig” (Figure 1). The populations of *Salmonella* on the leaf surface of the 3 cultivars one day after guttation inoculation were not significantly different (P = 0.264), while the populations inside the leaves of the 3 cultivars were significantly different (P = 0.049, Table 1). The linear model used to describe survival of *Salmonella* in each sample had a coefficient of variation (R^2^) of 0.921±0.091. With respect to estimates of initial bacterial concentration (intercept) and decline rate (slope), the *Salmonella* levels on/in the 3 tomato cultivars were significantly different, with an overall Wilk’s Lambda significance value less than 0.001. The levels of *Salmonella* on/in tomato leaves decreased fastest on cultivar “Florida Lanai” and slowest on cultivar “Ailsa Craig” (Fig. 1, Table 2). The intercepts of *Salmonella* populations were not significantly different on the surface of these 3 tomato cultivars, while the internal populations in the 3 cultivars were significantly different (Table 2). These results indicated that the entrance probability and survival of *Salmonella* in the tomato cultivars were different at a guttation inoculum concentration of 10^9^ CFU/ml.

**Table 1:** *Salmonella* Typhimurium population density (mean ± standard deviation) on/in tomato leaves 1 day after inoculation through guttation droplets (1 droplet per leaf).

Inoculation concentration	Position	Tomato cultivars	*Salmonella* population (log (CFU/g))
		Florida Lanai	2.236±1.099^a^
	Surface	Crown Jewel	3.168±0.513^a^
10^9^ CFU/ml		Ailsa Craig	3.337±0.644^a^
		Florida Lanai	1.735±1.502^b^
	Internal	Crown Jewel	3.602±0.301^a^
		Ailsa Craig	3.751±0.298^a^
		Florida Lanai	1.735±1.502^a^
	Surface	Crown Jewel	1.735±1.502^a^
10^7 ^CFU/ml		Ailsa Craig	0.926±0.807^a^
		Florida Lanai	0.000±0.000^a^
	Internal	Crown Jewel	1.369±1.195^a^
		Ailsa Craig	0.634±0.605^a^

a and ^b^indicate significant differences among cultivars using the least significant difference test of ANOVA (P = 0.05).

**Table 2:** Statistical analysis of parameter estimates for the decline of *Salmonella* Typhimurium concentrations on/in tomato leaves after inoculation through guttation droplets.

Inoculation concentration	Position	Tomato cultivars	Intercept (log (CFU/g))	Slope (log (CFU/g)·day^−1^)
		Florida Lanai	6.681±0.648^a^	−3.541±0.189^a^
	Surface	Crown Jewel	6.409±0.469^a^	−2.173±0.107^b^
10^9^ CFU/ml		Ailsa Craig	6.175±0.293^a^	−1.752±0.089^c^
		Florida Lanai	4.460±0.039^a^	−2.320±0.253^a^
	Internal	Crown Jewel	5.803±0.058^c^	−1.877±0.043^b^
		Ailsa Craig	5.045±0.499^b^	−1.292±0.251^c^
		Florida Lanai	4.264±0.921^a^	−2.204±0.294^a^
	Surface	Crown Jewel	4.293±0.958^a^	−2.221±0.321^a^
10^7 ^CFU/ml		Ailsa Craig	4.151±0.549^a^	−2.285±0.181^a^
		Florida Lanai	2.136±0.648^a^	−1.262±0.253^a^
	Internal	Crown Jewel	3.487±0.581^a^	−1.812±0.308^a^
		Ailsa Craig	2.677±0.278^a^	−1.461±0.076^a^

a , ^b^and^ c^indicate significant differences among cultivars using the least significant difference test of ANOVA (P = 0.05).

At the inoculum concentration of 10^7^ CFU/ml, *Salmonella* on/in tomato leaves could not be detected 2 days after inoculation for all 3 tomato cultivars (Figure 2). The populations of *Salmonella* on the leaf surface or inside the leaves of the 3 cultivars one day after guttation inoculation were not significantly different (surface: P = 0.699, internal: P = 0.176, Table 1). Based on MANOVA analysis, the decline in *Salmonella* concentrations on/in the 3 tomato cultivars were not significantly different, with an overall Wilk’s Lambda significance values of 0.477 (surface) and 0.428 (internal), respectively. The decline rate and initial population of *Salmonella* on/in tomato leaves of the 3 cultivars were also not significant (P>0.05, Table 2).

Different from the direct plating results, *Salmonella* was recovered by enrichment for 7 days after guttation inoculation at both inoculum concentrations for all 3 tomato cultivars.

### Effects of ABA and Guttation Production on the Internalization and Survival of *Salmonella* Typhimurium on/in Tomato Leaves through Guttation Inoculation

To analyze the effects of the plant hormone ABA and increase of guttation production on *Salmonella* entrance and persistence, the population density and survival trend of *Salmonella* Typhimurium on/in tomato leaves of the transgenic line Sp5 were compared with those of its wild type tomato cultivar “Ailsa Craig”. Similar to observations on “Ailsa Craig”, *Salmonella* populations on/in Sp5 declined after guttation inoculation. However, the detection period using the direct plate counting method was extended for 2 days for *Salmonella* on the leaf surface and 1 day for the internally colonized *Salmonella* in the transgenic plants after inoculation with 10^9^ CFU/ml (Fig. 1). The detection period for *Salmonella* on/in tomato leaves of “Ailsa Craig” and Sp5 were the same after inoculation with 10^7^ CFU/ml (Fig. 2).

There were significant interactions between plant genotypes (wild type “Ailsa Craig” and its transgenic line Sp5) and inoculation concentrations for both *Salmonella* populations 1 day after inoculation and for their decline rates on/in tomato leaves (P<0.05). At the inoculation concentration of 10^9^ CFU/ml, the population of *Salmonella* on the of leaf surface of Sp5 1 day after guttation inoculation was significantly higher than that of its wild type plants (P = 0.018), while the populations inside the leaves of the 2 tomato genotypes were not significantly different (P = 0.119). Based on the analysis of the parameters of the linear model, the *Salmonella* decline curves on the leaf surface or inside leaves were significantly different between the two genotypes, with an overall Wilk’s Lambda significance values of 0.003 and 0.001, respectively. The decline rate of *Salmonella* on the leaf surface of Sp5 was significantly higher than that of its wild type “Ailsa Craig” (P = 0.005), while there was no significant difference for the internal decline rate (P = 0.443). The intercepts of *Salmonella* on/in tomato leaves of Sp5 were significantly higher than those of “Ailsa Craig” (surface: P = 0.018; internal: P = 0.044).

At the inoculum concentration of 10^7^ CFU/ml, the populations of *Salmonella* on the leaf surface or inside the leaves of the 2 tomato genotypes 1 day after guttation inoculation were not significantly different (surface: P = 0.458, internal: P = 0.271). Based on MANOVA analysis, the *Salmonella* decline curves were not significantly different for the two genotypes, with overall Wilk’s Lambda significance values of 0.751 and 0.350, for surface and internal populations respectively. The decline rate and initial population of *Salmonella* on/in tomato leaves of the 2 tomato genotypes were also not significantly different (P>0.05).

*Salmonella* could still be recovered by enrichment 7 days after guttation inoculation at both inoculum concentrations from the transgenic Sp5 as well as from the wild type.

### Microscopic Detection of *Salmonella* Ingress into Tomato Leaves through Hydathodes

**Figure 3:**
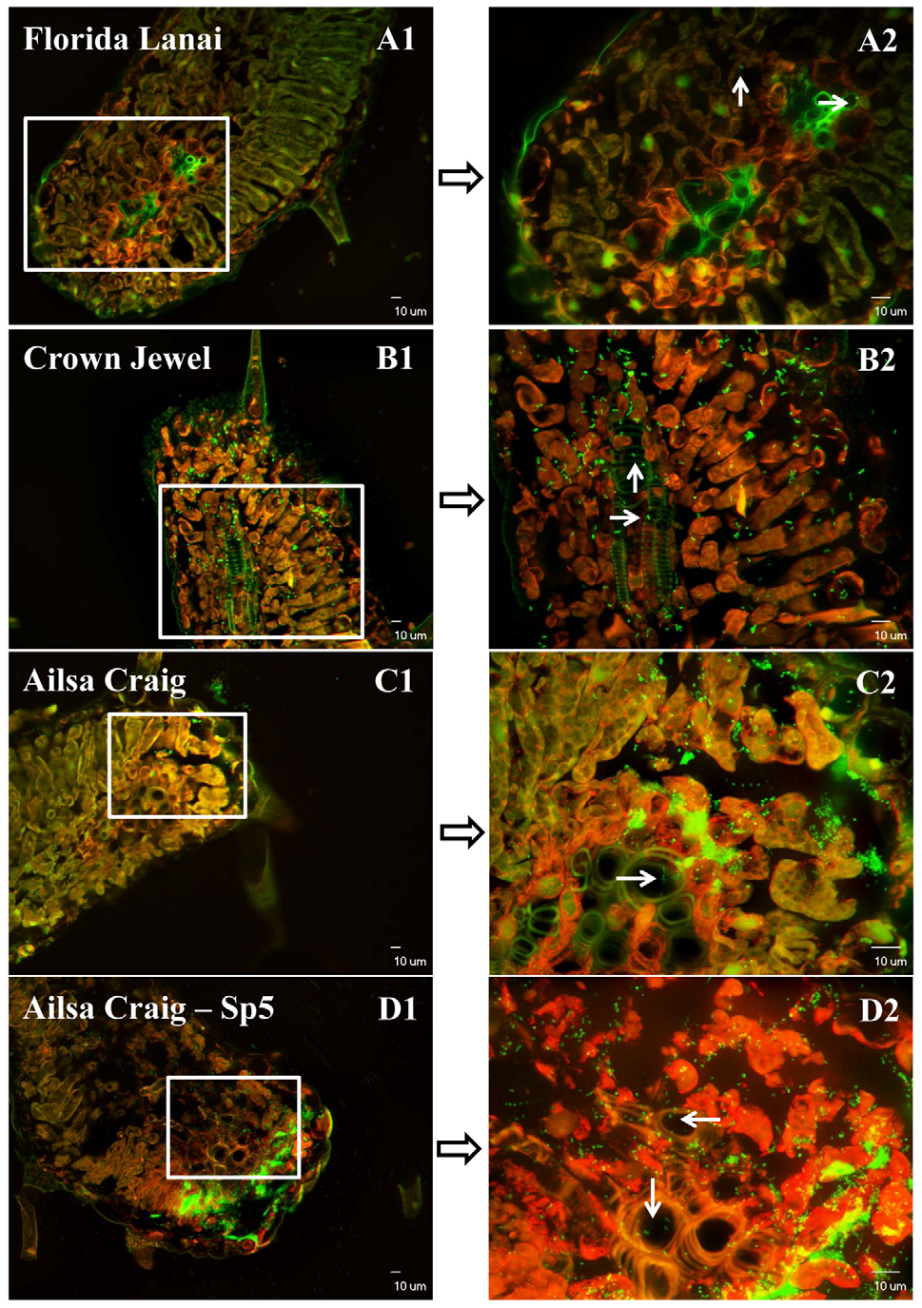
Confocal laser microscope images of tomato leaf tissue sections colonized by *Salmonella* Typhimurium after inoculation (10^9^ CFU/ml) through guttation droplets. White arrows point out the GFP-tagged *Salmonella* cells (green) which entered into the vascular system of tomato leaves. Red fluorescence is the autofluorescence of plant chloroplasts. Images A2, B2, C2 and D2 are merged images under GFP and TRITC filters obtained by projecting 20 Z section overlaid fluorescence images of different layers with 1 µm interval into one combined image [11].

The entrance of *Salmonella* Typhimurium into tomato leaves through guttation droplets was confirmed under the confocal laser microscope (Fig. 3). All the 6 inoculated leaflets of each genotype of tomato plants were *Salmonella* positive (about 50% of the leaf sections for each inoculated leaflet were positive). One day after inoculating *Salmonella* cells into the guttation droplets (Fig. S1), the bacteria entered into the tips of the tomato leaves of all the 4 genotypes of tomato plants, and some of the cells moved into the vascular systems. The density of GFP labeled *Salmonella* cells in the inoculated leaves of the 4 genotypes of tomato plants matched the survival trend of this bacterium inside the plants: Sp5> “Ailsa Craig”>“Crown Jewel”>“Florida Lanai”. All these microscopic results supported the ingress of *S. enterica* Typhimurium into tomato leaves through hydathodes. As expected, *Salmonella* cells were not detected in leaf samples from control plants (Fig. S2).

## Discussion

Our study shows, for the first time, that *S. enterica* Typhimurium can enter tomato leaf tips through hydathodes, and move into the vascular system after guttation inoculation, providing a possibility for internal translocation of *Salmonella* cells in the plants. While there are dense cell layers between stomatal cavities and the vascular system, hydathodes are directly linked to the vascular system in plant leaves. And this system might be the main entrance site for the internalization of this human pathogen into plants. When internalization and transport of *Salmonella* through the phloem was observed [11], leaf-dip inoculation had taken place in Winter, under conditions of frequent natural guttation in the greenhouse. When leaf-dip inoculation was carried out at other times of year, internalization and translocation to fruits usually failed (unpublished results). Even though *Salmonella* cells were observed inside stomates of Winter-inoculated leaves [11], the main entry way into the vascular system may have been the hydathodes.

Entrance and internal persistence of *Salmonella* in tomato leaves were affected by tomato cultivars after guttation inoculation. The bacterial cells survived best in a commercial cultivar “Ailsa Craig”, and decreased most quickly in a non-commercial cultivar “Florida Lanai”, which was used in previous studies [11], [26]. However, the cultivar effect was only significant after inoculation through guttation droplets with a suspension of 10^9^ CFU/ml, amounting to 10^8^ CFU/ml of guttation fluid. *Salmonella* populations and survival trends were not significantly different for the 3 tomato cultivars at the inoculation concentration of 10^7^ CFU/ml (10^6^ CFU/ml of guttation fluid). These results indicated that the inoculation amount might affect the survival of *Salmonella* on/in tomato leaves and the interaction of *Salmonella* Typhimurium with different tomato cultivars. Another probability is that the sample size may not be large enough to detect a statistically significant difference at the lower inoculum concentration. Further studies with large sampling sizes (>100 plants) will be needed to analyze the cultivar effect on *Salmonella* survival at lower inoculum concentrations.

*Salmonella* survived longer on/in the tomato leaves of the transgenic line Sp5 compared to its wild type “Ailsa Craig”. The initial population (intercept) of *Salmonella* on/in tomato leaves was significantly higher on/in Sp5 plants. However, the bacterial population 1 day post inoculation (at 10^9^ CFU/ml) and the decline rate were only significantly different for the survival of *Salmonella* on the surface of tomato leaves. The main hypothesis is that the over production of guttation increased the humidity and exuded nutrients at the tips of tomato leaves, which may have benefited the survival of the bacteria on the surface. Nevertheless, the production of ABA did not seem to be associated with plant resistance or susceptibility to this human pathogen.

Even though *Salmonella* could not be detected 5 days after inoculation from all of the tomato plants at both inoculation concentrations by the direct plating method, the bacterial cells could still be recovered from the inoculated plants 1 week later. These results indicated that this human pathogen might survive for a long period on plants at a low population density. Moreover, if only a few cells could reach tomato fruits through the vascular system, these could multiply within the fruits to densities that could provide a risk to consumers [11].

Another interesting observation in this study was that the *Salmonella* population was increased 1 day after inoculation instead of decreased in one weak, slow growing “Crown Jewel” plant, where *Pythium* was isolated from the roots (Fig. S3). Similar as reported before [27], the extensive multiplication of *Salmonella* Typhimurium inside leaves of this plant indicated that infection by a plant pathogen might benefit the survival and even multiplication of *Salmonella* in tomato plants. Further studies will need to be conducted to study the interactions of plant pathogens and human pathogens on plants.

## Supporting Information

Figure S1 **Guttation droplets at the tips of leaves of different tomato genotypes in a BSL2 growth chamber.** Florida Lanai (A), Crown Jewel (B), Ailsa Craig (C) and the transgenic line (Sp5) of Ailsa Craig (D).
(TIF)

Figure S2 **Confocal microscopy of leaf tips of control plants (without** ***Salmonella*** **inoculation).** The white arrows point out the hydathodes at the tips of tomato leaves of cultivar Ailsa Craig (A) and the transgenic line (Sp5) of Ailsa Craig (B).
(TIF)

Figure S3 **Confocal microscopy of** ***Salmonella*** **Typhimurium in leaf tissue sections from one weak tomato plant of Crown Jewel after guttation inoculation.** A1/A2 and B1/B2 are different leaf sections of the same tomato plant. *Pythium* sp. was isolated from the root of this weak plant (C). Images A2 and B2 are merged images under GFP and TRITC filters obtained by projecting 20 Z section overlaid fluorescence images of different layers with 1 µm interval into one combined image.
(TIF)
