# Supplementary figures and images for: Ingress of Salmonella enterica Typhimurium into Tomato Leaves through Hydathodes
Source: PLoS One. 2013 Jan 8;8(1):e53470. doi: 10.1371/journal.pone.0053470 (PMC3540056; doi:10.1371/journal.pone.0053470)

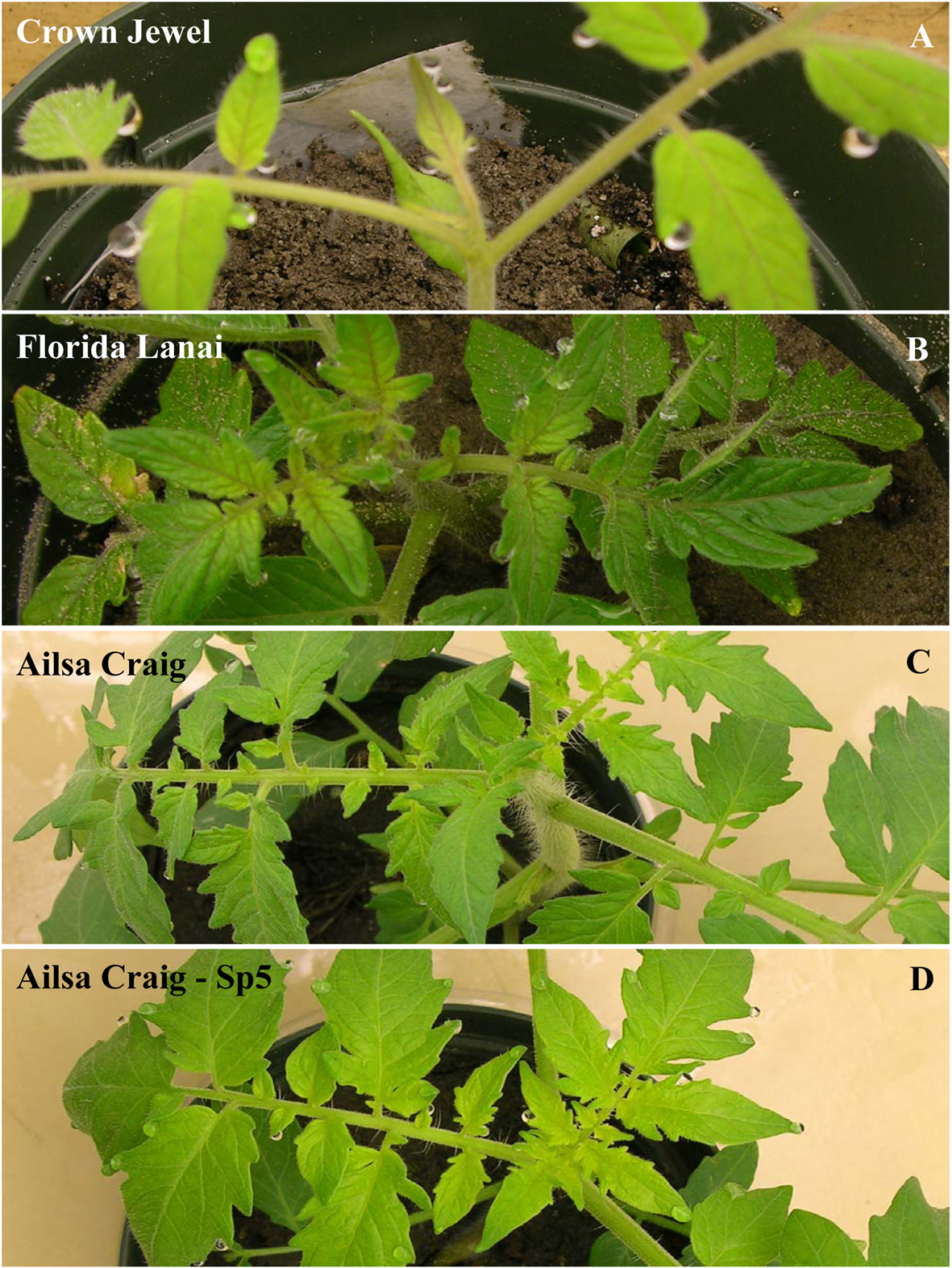

Supplement: Figure S1 — Guttation droplets at the tips of leaves of different tomato genotypes in a BSL2 growth chamber. Florida Lanai (A), Crown Jewel (B), Ailsa Craig (C) and the transgenic line (Sp5) of Ailsa Craig (D). (TIF) [file pone.0053470.s001.tif]

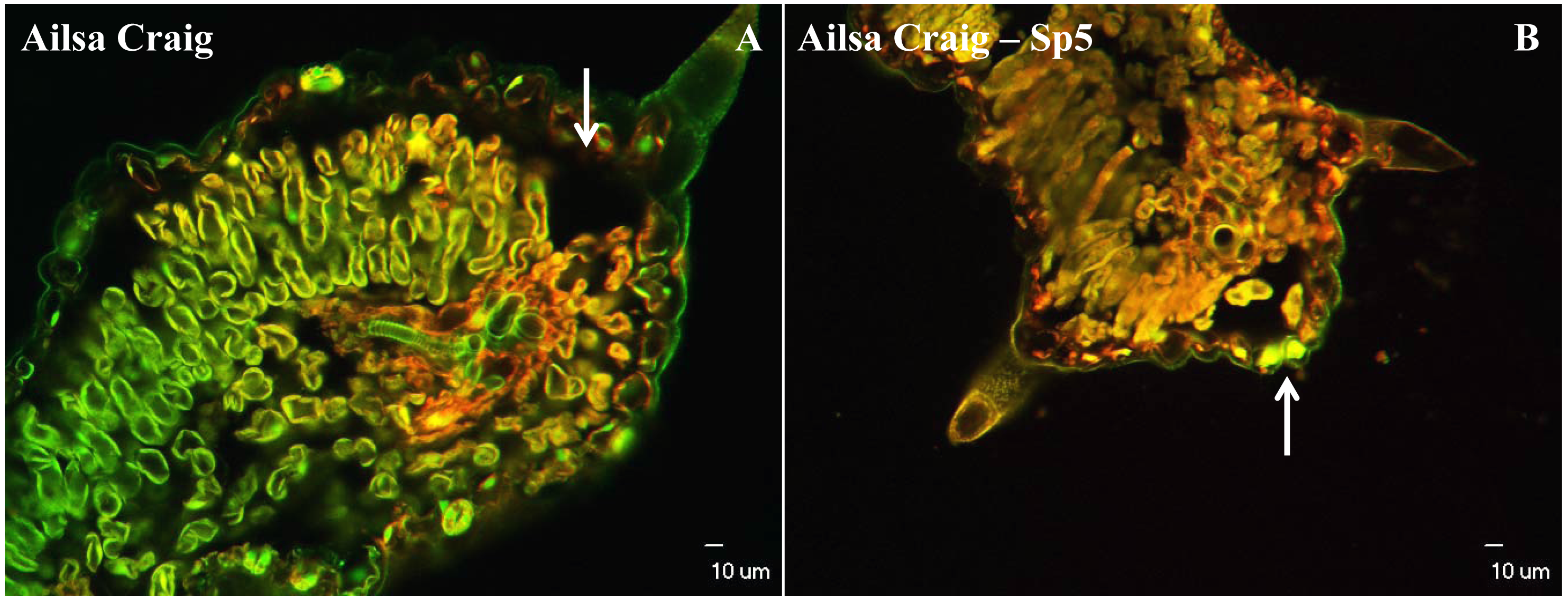

Supplement: Figure S2 — Confocal microscopy of leaf tips of control plants (without Salmonella inoculation). The white arrows point out the hydathodes at the tips of tomato leaves of cultivar Ailsa Craig (A) and the transgenic line (Sp5) of Ailsa Craig (B). (TIF) [file pone.0053470.s002.tif]

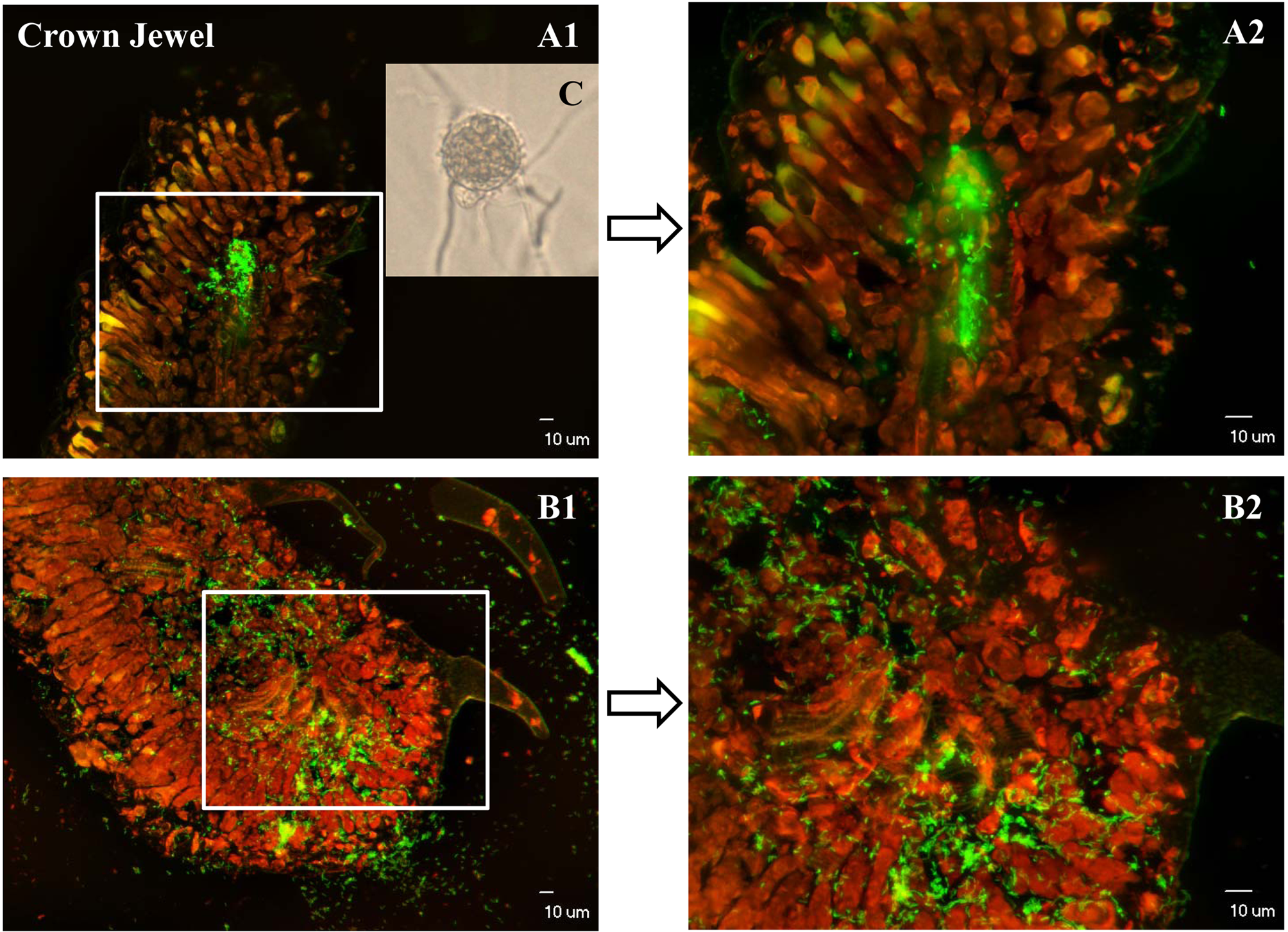

Supplement: Figure S3 — Confocal microscopy of Salmonella Typhimurium in leaf tissue sections from one weak tomato plant of Crown Jewel after guttation inoculation. A1/A2 and B1/B2 are different leaf sections of the same tomato plant. Pythium sp. was isolated from the root of this weak plant (C). Images A2 and B2 are merged images under GFP and TRITC filters obtained by projecting 20 Z section overlaid fluorescence images of different layers with 1 µm interval into one combined image. (TIF) [file pone.0053470.s003.tif]
